# Uncovering the candidate genes related to sheep body weight using multi-trait genome-wide association analysis

**DOI:** 10.3389/fvets.2023.1206383

**Published:** 2023-08-17

**Authors:** Yunna Li, Hua Yang, Jing Guo, Yonglin Yang, Qian Yu, Yuanyuan Guo, Chaoxin Zhang, Zhipeng Wang, Peng Zuo

**Affiliations:** ^1^College of Animal Science and Technology, Northeast Agricultural University,, Harbin, China; ^2^State Key Laboratory of Sheep Genetic Improvement and Healthy Production, Xinjiang Academy of Agricultural and Reclamation Science,, Shihezi, China; ^3^College of Science, Northeast Agricultural University, Harbin, China

**Keywords:** sheep, body weight, genome-wide association analysis, multi-trait, single-trait

## Abstract

In sheep, body weight is an economically important trait. This study sought to map genetic loci related to weaning weight and yearling weight. To this end, a single-trait and multi-trait genome-wide association study (GWAS) was performed using a high-density 600 K single nucleotide polymorphism (SNP) chip. The results showed that 43 and 56 SNPs were significantly associated with weaning weight and yearling weight, respectively. A region associated with both weaning and yearling traits (OARX: 6.74–7.04 Mb) was identified, suggesting that the same genes could play a role in regulating both these traits. This region was found to contain three genes (*TBL1X, SHROOM2* and *GPR143*). The most significant SNP was Affx-281066395, located at 6.94 Mb (*p* = 1.70 × 10^−17^), corresponding to the *SHROOM2* gene. We also identified 93 novel SNPs elated to sheep weight using multi-trait GWAS analysis. A new genomic region (OAR10: 76.04–77.23 Mb) with 22 significant SNPs were discovered. Combining transcriptomic data from multiple tissues and genomic data in sheep, we found the *HINT1*, *ASB11* and *GPR143* genes may involve in sheep body weight. So, multi-omic anlaysis is a valuable strategy identifying candidate genes related to body weight.

## 1. Introduction

GWAS have been widely used in gene mapping research to understand the genetic mechanisms governing economically important traits in sheep, including weight, reproductive fitness, horn number, ear type, hair color, and disease resistance (1–6). The first study to use this approach in sheep focused on horn shape and revealed that the *RXFP2* gene is related to horn type in sheep (7).

Body weight is the most important index of growth and development in farmed sheep. Studies have noted heritabilities of 0.30–0.35 for weaning weight and 0.40–0.45 for yearling weight, indicating that the heritability of these traits is moderately (8). Based on GWAS, Gholizadeh et al. (9) who used the Illumina ovine SNP50 BeadChip in 96 Baluchi sheep discovered the candidate genes *TRBP* and *TRAMIL1* for birth weight; *APIP* and *DAAM1* for weaning weight; *PHF15, PRSS12,* and *MAN1A1* for 6-month weight; and *SYNE1, WAPAL*, and *DAAM1* for yearling weight. Al-Mamun et al. (10) used data from 1781 Australian Merino sheep genotyped with the Illumina Ovine SNP 50 K BeadChip and found that the genes *LAP3, NCAPG*, and *LCORL* are related to body weight traits in sheep. Similarly, using GWAS, Ghasemi et al. (11) demonstrated that *RAB6B* and *GIGYF2* are candidate genes for birth weight using Illumina Ovine SNP50 Bead Chip from 132 Lori-Bakhtiari sheep, and Lu et al. (1) performed a genome-wide associations of birth, weaning, yearling, and adult weights of 460 fine-wool sheep were determined using resequencing technology. The results showed that 113 single nucleotide polymorphisms (SNPs) reached the genome-wide significance levels for the four body weight traits and 30 genes were annotated effectively, including *AADACL3, VGF, NPC1,* and *SERPINA12.*

When traits are highly correlated with each other, multi-trait analysis is more advantageous than single-trait analysis. Because multi-trait GWAS involves only one statistical test and considers both the intra- and inter-trait variance components of multiple traits (12), it can reduce the errors caused by multiple testing (13). Hence, it improves the accuracy (14, 15) and precision of parameter estimation (16). Multi-trait GWAS also increases the statistical power by exploiting the genetic correlation between different traits.

In this study, we used the Affymetrix Ovis600K genotyping bead chip to identify candidate genes related to weaning weight and yearling weight using multi-trait and single-trait GWAS. Our findings provide a reference for understanding the inheritance mechanism of weight traits in sheep.

## 2. Materials and methods

### 2.1. Ethics statement

All experimental procedures were in accordance with animal welfare legislation and were approved by the Experimental Animal Care and Use Committee of Xinjiang Academy of Agricultural and Reclamation Sciences (Shihezi, China, Ethics committee approval number: XJNKKXY-2020-34; December 30, 2020).

### 2.2. Sample collection and genotyping

A total of 218 ewes (a composite line bred from Australian Suffolk sheep, Chinese Hu sheep, and Chinese Kazakh sheep) were collected from the Xinjiang Academy of Agricultural and Reclamation Science. We recorded their weights at two stages: weaning and yearling. All sheep were fasted for 12 h before their weaning weights and first yearling weights were measured.

Single nucleotide polymorphisms (SNPs) were examined using the Affymetrix Ovis600K genotyping bead chip, which contains 604,721 SNPs. Plink 1.9 software (17) was used to control the quality of genotype data; (1) minor allele frequency (MAF) ≥5% SNPs, (2) SNP call rate ≥ 95%, (3) individual call rate ≥ 90%, and (4) SNPs mapped to X chromosomes and autosomes were evaluated. After the quality control was performed on the raw genotypes, a total of 218 animals and 479,470 SNPs were obtained. In this study, Beagle software was used to fill in the missing genotypes. The Haploview software (18) was used to analyze the linkage disequilibrium (LD). The haplotype block recognition algorithm proposed by Gabriel et al. (19), their criterion is that the one-sided upper 95% confidence bound on D′ is >0.98 and the lower bound is >0.70.

### 2.3. Estimation of genetic parameters of weaning weight and yearling weight

GCTA was developed as a method for estimation the variance explained by all the SNPs on a chromosome or on the whole genome for a complex trait (20). In this study, −-reml and −-reml-bivar were set to calculate heritability and genetic correlation, respectively. Using SAS9.4 (21), descriptive statistics were performed for these two traits, including mean, standard deviations, coefficients of variation.

### 2.4. Single-trait and multi-trait GWAS

In this study, a mixed linear model was used to analyze the association between SNPs and body weight traits, including weaning weight and yearling weight. The model used was as follows:


Y=μ+Xb+∑Kp+Ms+Za+Qc+e,

where Y is the phenotype value vector, b is the fixed effect vector (year effect), p is the top three eigenvectors of principal component analysis (PCA), s is the SNP effect vector and SNP genotypes coded as 0, 1 and 2 for aa, Aa and AA, a is the individual residual polygene effect (random effect), c is the birth weight vector (covariant), e is the random residual effect vector, and X, K, Z, Q are the design matrices of b, p, s, a, and c, respectively.

Multivariate mixed linear models (mvLMMs) were used to conduct joint association analysis between SNPs and two traits due to strong genetic correlation between weaning weight and yearling weight.

GEMMA software (16) was used to perform single-trait and bi-trait GWAS. The Wald test was used to evaluate the significance of each genetic marker. In order to reduce false positives, the Bonferroni correction method was applied correction, and the threshold value was *p* = 0.05/479470 = 1.04 × 10^−7^ (single-trait) and *p* = 0.05/479470/2 = 5.70 × 10^−8^ (bi-trait).

### 2.5. Function annotations

In this study, we first downloaded the sheep *Ovis aries* (Oar_v3.1) gene annotation information^1^ from the Ensembl database (22), then used the intersect parameter of BEDTools 2.1.2 software to annotatin the significant SNPs and identifying gene within 200 kb upstream and downsteam of these SNPs (23). We collected quantitative trait loci (QTLs) related to sheep weight traits from the animal QTL database.^2^

### 2.6. Expression analysis

We collected RNA-Seq data from the European Bioinformatics Institute (EBI) for 17 tissues, including muscle long dorsal, muscle biceps, spleen, lung, pituitary gland, brain, hypothallamus, mammary gland, kidney cortex, kidney medulla, heart, rectum, abomasum, uterus, colon, rumen, ovary tissues of juvenile and adult sheep (BioProject number: PRJEB6169). There are more than 3 samples each tissues of juvenile or adult sheep. After using Trimmomatic to remove adapter and low-quality sequences (24), all RNA-Seq datasets were processed using FastQC v0.11.3^3^ and quality inspection was conducted. Reads were aligned to the sheep reference genome (OARv3.1) using STAR v.2.7.6a (25) and counted with the RNA-Seq by Expectation Maximization (RSEM) software v. 1.3.3 (26). The DESeq2 package in R was used to DEGs with significant differences between different samples (27). The tissue specificity index (τ) of the candidate genes was calculated, which is defined as


$$\tau =\frac{\sum_{i=1}^{N}\left(1-x_{i}\right)}{N-1}$$

where $x_{i}$ is the expression profile component normalized by the maximal component value and N is the number of tissues (28).

## 3. Results

### 3.1. Descriptive statistics and genetic parameters

In this study, the descriptive statistics of weaning weight and yearling weight traits were analyzed including mean, standard deviations, coefficients of variation (Table 1). Based on SNP genotype, we used the GCTA software calculate genetic parameter of weaning and yearling weight traits. We found the heritability of weaning weight and yearling weight was 0.54 and 0.44, respectively. There was a significant positive genetic correlation between weaning weight and yearling weight with a correlation coefficient of 0.73 (*p* < 0.05).

**Table 1:** Descriptive statistics of body weight.

Trait	Mean	Standard deviation (SD)	Maximum value	Minimum value	Coefficient of variation (%)
Weaning weight (kg)	23.74	4.69	30.50	15.00	19.76
Yearling weight (kg)	40.89	8.03	62.20	28.00	19.64

### 3.2. Single-trait GWAS

**Figure 1:**
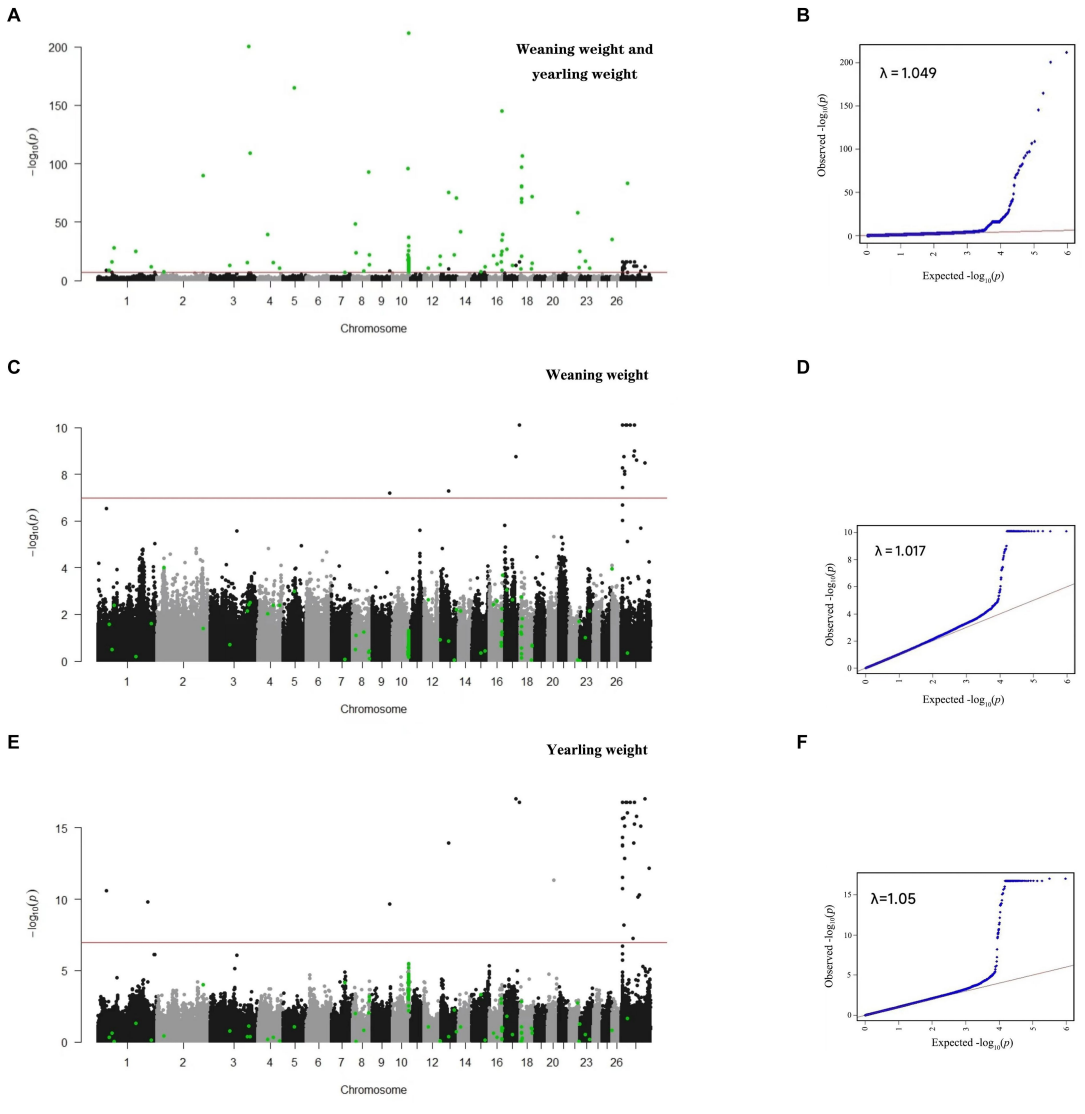
Manhattan and QQ plots for single- and multi-trait GWAS. Multi-trait **(A,B)**; weaning weight **(C,D)**; and yearling weight **(E,F)**.

In this study, single-trait GWAS was conducted for weaning weight and yearling weight based on a linear mixed model. We identified 43 and 56 SNPs significantly related to weaning weight and yearling weight (Table 2), respectively. The corresponding Manhattan and quantile-quantile (Q-Q) plots are shown in Figure 1. For weaning weight, significantly SNPs were detected on the chromosomes OAR9, OAR13, OAR17 and OARX. These SNPs were located nearest to the genes including *ESRP1* (Epithelial Splicing Regulatory Protein 1), *MPP7* (MAGUK P55 Scaffold Protein 7), *WDR66* (WD Repeat-containing protein 66), *SHROOM2* (Shroom Family Member 2). For yearling weight, significantly SNPs were located on OAR1, OAR9, OAR13, OAR17, OAR20 and OARX. The following genes are annotated *FOXD3* (Forkhead box D3), *ESRP1, MPP7, WDR66, DOCK11* (Dedicator Of Cytokinesis 11).

**Table 2:** Significant loci and genes identified using single-trait GWAS.

Trait	Top-SNP	Chromosome	Position (Mb)	Beta	SE	*p* value	Candidate gene	Distance (kb)
Weaning weight	Affx-280934168	9	82.30	−12.41	2.17	6.21 × 10^−8^	*ESRP1*	6.70
	Affx-281160655	13	35.58	15.36	2.67	5.17 × 10^−8^	*MPP7*	Intron
	Affx-280817808	17	52.92	18.32	2.84	1.78 × 10^−9^	*WDR66*	13.79
	Affx-280850001	17	70.28	20.70	2.94	8.09 × 10^−11^	*IGLV4-60*	Intron
	Affx-281066395	X	6.94	20.70	2.94	8.09 × 10^−11^	*SHROOM2*	Intron
	Affx-280971786	X	13.58	18.32	2.84	1.78 × 10^−9^	*GRPR*	Intron
	Affx-280750675	X	14.69	14.82	2.43	1.01 × 10^−8^	*REPS2*	26.57
	Affx-281271848	X	15.57	16.31	2.65	7.65 × 10^−9^	*HMGA2*	117.45
	Affx-281246794	X	21.47	20.70	2.94	8.09 × 10^−11^	*ZFX*	Exon
	Affx-281100183	X	25.79	20.70	2.94	8.09 × 10^−11^	–	–
	Affx-280837435	X	40.90	20.70	2.94	8.09 × 10^−11^	*NDP*	85.12
	Affx-280944812	X	59.02	16.87	2.61	1.66 × 10^−9^	*FAM155B*	1.11
	Affx-280773186	X	60.72	18.25	2.79	1.00 × 10^−9^	*TAF1*	Intron
	Affx-280784773	X	61.24	20.70	2.94	8.09 × 10^−11^	*RTL5*	79.14
	Affx-281227833	X	70.46	18.25	2.86	2.51 × 10^−9^	*POU3F4*	149.90
	Affx-281119406	X	110.63	16.19	2.56	3.32 × 10^−9^	*DOCK11*	Intron
Yearling weight	Affx-281233109	1	38.36	17.11	2.40	2.61 × 10^−11^	*FOXD3*	1.88
	Affx-281153321	1	232.51	16.37	2.41	1.62 × 10^−10^	*THOC2*	Intron
	Affx-280934168	9	82.30	12.94	1.92	2.28 × 10^−10^	*ESRP1*	6.70
	Affx-281160655	13	35.58	19.15	2.27	1.19 × 10^−14^	*MPP7*	Intron
	Affx-280817808	17	52.92	21.67	2.26	9.10 × 10^−18^	*WDR66*	13.79
	Affx-280850001	17	70.28	21.68	2.28	1.70 × 10^−17^	*IGLV4-60*	Intron
	Affx-280818224	20	30.99	13.12	1.77	4.76 × 10^−12^	*SLC17A1*	1.59
	Affx-280871230	X	6.72	13.15	1.75	3.01 × 10^−12^	*TBL1X*	0.04
	Affx-281066395	X	6.94	21.68	2.28	1.70 × 10^−17^	*SHROOM2*	Intron
	Affx-281021986	X	12.75	9.75	1.59	6.27 × 10^−9^	*ASB11*	Exon
	Affx-280971786	X	13.58	21.14	2.32	1.96 × 10^−16^	*GRPR*	Intron
	Affx-280750675	X	14.69	18.61	2.32	1.40 × 10^−13^	*REPS2*	26.57
	Affx-281271848	X	15.57	20.80	2.34	8.07 × 10^−16^	*HMGA2*	117.45
	Affx-281246794	X	21.47	21.68	2.28	1.70 × 10^−17^	*ZFX*	Exon
	Affx-281100183	X	25.79	21.68	2.28	1.70 × 10^−17^	–	–
	Affx-280975370	X	27.47	21.84	2.37	9.16 × 10^−17^	*MAGEB2*	5.58
	Affx-280837435	X	40.90	21.68	2.28	1.70 × 10^−17^	*NDP*	85.12
	Affx-280901032	X	53.35	9.33	1.64	5.67 × 10^−8^	*SSX2*	3.48
	Affx-280944812	X	59.02	19.44	2.30	1.11 × 10^−14^	*FAM155B*	1.11
	Affx-280773186	X	60.72	20.90	2.34	5.74 × 10^−16^	*TAF1*	Intron
	Affx-280784773	X	61.24	21.68	2.28	1.70 × 10^−17^	*RTL5*	79.14
	Affx-281227833	X	70.46	20.98	2.29	1.63 × 10^−16^	*POU3F4*	149.90
	Affx-281174947	X	78.06	16.04	2.31	7.09 × 10^−11^	*ZNF517*	40.95
	Affx-281019020	X	84.68	18.29	2.61	4.95 × 10^−11^	*SLITRK2*	Exon
	Affx-280983473	X	92.32	18.55	2.08	7.60 × 10^−16^	–	–
	Affx-281119406	X	110.63	21.15	2.20	9.18E × 10^−18^	*DOCK11*	Intron
	Affx-281112347	X	129.06	15.83	2.04	6.93 × 10^−13^	–	–

**Figure 2:**
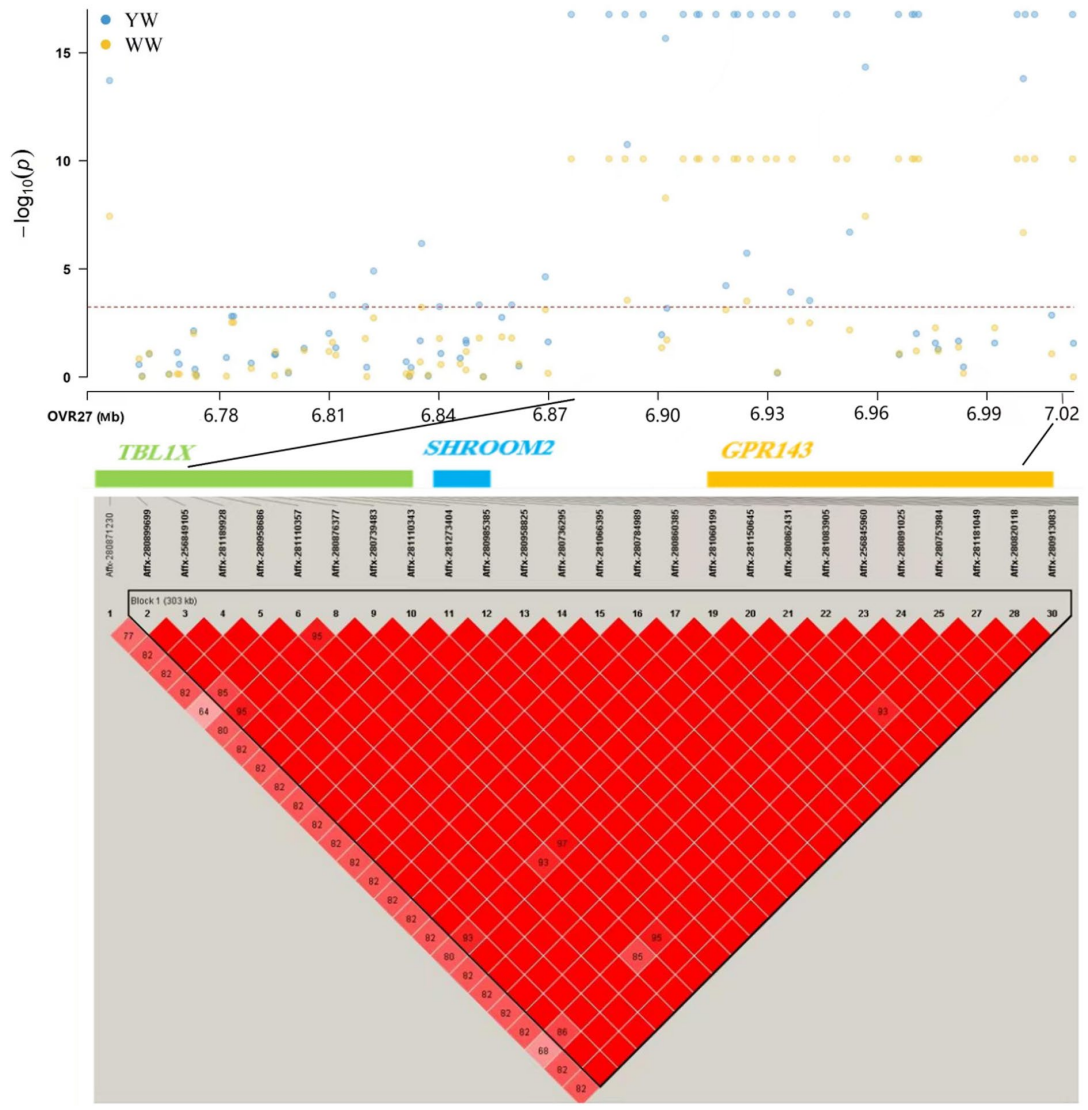
Association mapping results for SNPs significantly related to weaning weight and yearling weight located at 6.74–7.04 Mb on chromosome X. YW: yearling weight; WW: weaning weight.

In this study, 43 SNPs significantly associated with both traits (weaning weight and yearling weight), such as Affx-2809971786 (OARX: 13.58 Mb), Affx-281271848 (OARX: 15.57 Mb), and Afffx-281246794 (OARX: 21.47 Mb), located within the *GRPR* (Gastrin-releasing peptide receptor), *HMGA2* (High mobility group A 2), and *ZFX* (Zinc finger protein X-linked) genes, respectively. A 0.3-Mb region (6.74–7.04 Mb) on the X chromosome was the lagest region significantly associated with weaning weight and yearling weight (Figure 2). In this region, 27 and 30 SNPs were associated with weaning weight and yearling weight, respectively. These SNPs showed strong LD relationships with each other (*r*^2^ = 0.99). Moreover, this region contained the *TBL1X* (Transducing β-like 1 X-linked)*, GPR143* (G-protein coupled receptor143), and *SHROOM2* genes. The most significant SNP was Affx-281066395, located at 6.94 Mb (*P*_weaning weight_ = 8.09 × 10^−11^ and *P*_yearling weight_ = 1.70 × 10^−17^) on the *SHROOM2* gene. These findings suggest that this genetic region may have pleiotropic effects.

In addition, we also found two SNPs that were only related to yearling weight, i.e., Affx-281233109 and Affx-28153321.

### 3.3. Multi-trait GWAS results

Since there are significant highly genetic correlation between weaning weight and yearling weight, the multuvariate model is more accurate. We used bi-traits GWAS to identify novel significant SNPs. In this study, 138 SNPs related to sheep weight were identified, and the Q-Q and Manhattan plots are displayed in Figure 1. In contrast to single-trait GWAS, multi-trait GWAS identified 93 novel SNPs (Table 3), which were mainly located on five chromosomes, including OAR3, 8, 10, 16, and 18.

**Table 3:** Novel significant loci identified using multi-trait GWAS.

SNP	Chromosome	Position (Mb)	*p* value	Candidate gene	Distance (kb)
Affx-280755999	1	52.11	1.23 × 10^−9^	*ST6GALNAC3*	0.81
Affx-280791722	1	66.84	1.42 × 10^−16^	*ZNF326*	191.97
Affx-281090386	1	74.53	1.69 × 10^−28^	*ZCCHC17*	19.66
Affx-280867761	1	178.77	1.33 × 10^−25^	*LSAMP*	58.79
Affx-281226034	1	248.89	2.28 × 10^−12^	*NME9*	Intron
Affx-280821432	2	32.86	2.63 × 10^−8^	–	–
Affx-280856574	2	219.05	1.50 × 10^−90^	*TNS1*	Intron
Affx-281268994	3	93.11	1.27 × 10^−13^	*ZNF638*	Exon
Affx-281114490	3	175.87	2.71 × 10^−16^	*BTBD11*	25.21
Affx-122820109	3	181.88	4.54 × 10^−201^	*DNM1L*	Intron
Affx-281054367	3	187.97	1.66 × 10^−109^	*ITPR2*	Intron
Affx-280981488	4	47.91	2.34 × 10^−40^	*PIK3CG*	83.44
Affx-280901247	4	74.18	7.37 × 10^−16^	–	–
Affx-280776832	4	100.73	2.65 × 10^−11^	*PTN*	52.69
Affx-281218321	5	52.25	1.87 × 10^−165^	–	–
Affx-122856956	8	13.44	5.86 × 10^−49^	*RNF217*	117.55
Affx-280967487	8	15.58	2.55 × 10^−24^	*PKIB*	7.08
Affx-281261983	8	53.17	7.74 × 10^−9^	*THEMIS*	39.57
Affx-281048989	8	76.25	2.96 × 10^−93^	*MYCT1*	11.08
Affx-122857334	8	80.13	1.40 × 10^−22^	*ARID1B*	Intron
Affx-280965921	10	73.42	1.88 × 10^−96^	*HS6ST3*	11.93
Affx-281173612	10	74.53	8.96 × 10^−23^	*FARP1*	36.29
Affx-280892681	10	76.34	1.43 × 10^−212^	PCCA	Intron
Affx-122835917	10	76.85	9.22 × 10^−10^	*HINT1*	16.84
Affx-280950308	12	20.81	1.67 × 10^−11^	*LYPLAL1*	Intron
Affx-280946111	12	77.47	1.21 × 10^−21^	*CAMSAP2*	17.41
Affx-122843631	13	35.93	3.49 × 10^−76^	*MKX*	5.06
Affx-280906468	13	63.98	1.61 × 10^−22^	*EDEM2*	Intron
Affx-281176659	13	72.38	1.83 × 10^−71^	*HNF4A*	5.52
Affx-280976876	14	9.89	1.31 × 10^−42^	*MBTPS1*	Intron
Affx-280812512	15	41.44	1.98 × 10^−08^	*EIF4G2*	Intron
Affx-280868473	15	62.29	2.45 × 10^−12^	*KIAA1549L*	Intron
Affx-280962571	16	21.09	3.69 × 10^−22^	*PLK2*	177.834
Affx-280934775	16	36.13	8.59 × 10^−15^	*EGFLAM*	60.56
Affx-281116412	16	54.92	6.86 × 10^−17^	–	–
Affx-280870352	16	59.26	5.96 × 10^−146^	*DNAH5*	Intron
Affx-280741710	16	61.07	2.49 × 10^−40^	–	–
Affx-280771553	17	10.43	2.11 × 10^−27^	*EDNRA*	Intron
Affx-280903822	17	37.64	1.05 × 10^−13^	–	–
Affx-281077042	18	5.24	1.27 × 10^−10^	*CERS3*	Intron
Affx-281124791	18	8.24	6.34 × 10^−71^	–	–
Affx-281117901	18	9.28	2.26 × 10^−107^	–	–
Affx-280975510	18	53.86	8.22 × 10^−11^	*FANCM*	Intron
Affx-281012099	18	57.17	8.52 × 10^−73^	*UNC79*	Intron
Affx-280999698	22	40.15	8.67 × 10^−59^	*IL11*	74.71
Affx-281231501	22	45.21	4.45 × 10^−12^	*C10orf90*	99.50
Affx-280930203	22	50.00	1.26 × 10^−25^	*INPP5A*	53.989
Affx-122833966	23	24.82	1.80 × 10^−17^	*ERVW-1*	27.38
Affx-281156572	23	45.10	4.74 × 10^−11^	*SETBP1*	144.56
Affx-281178806	26	1.65	5.41 × 10^−36^	–	–
Affx-280838398	X	26.99	1.09 × 10^−83^	*IL1RAPL1*	104.56

**Figure 3:**
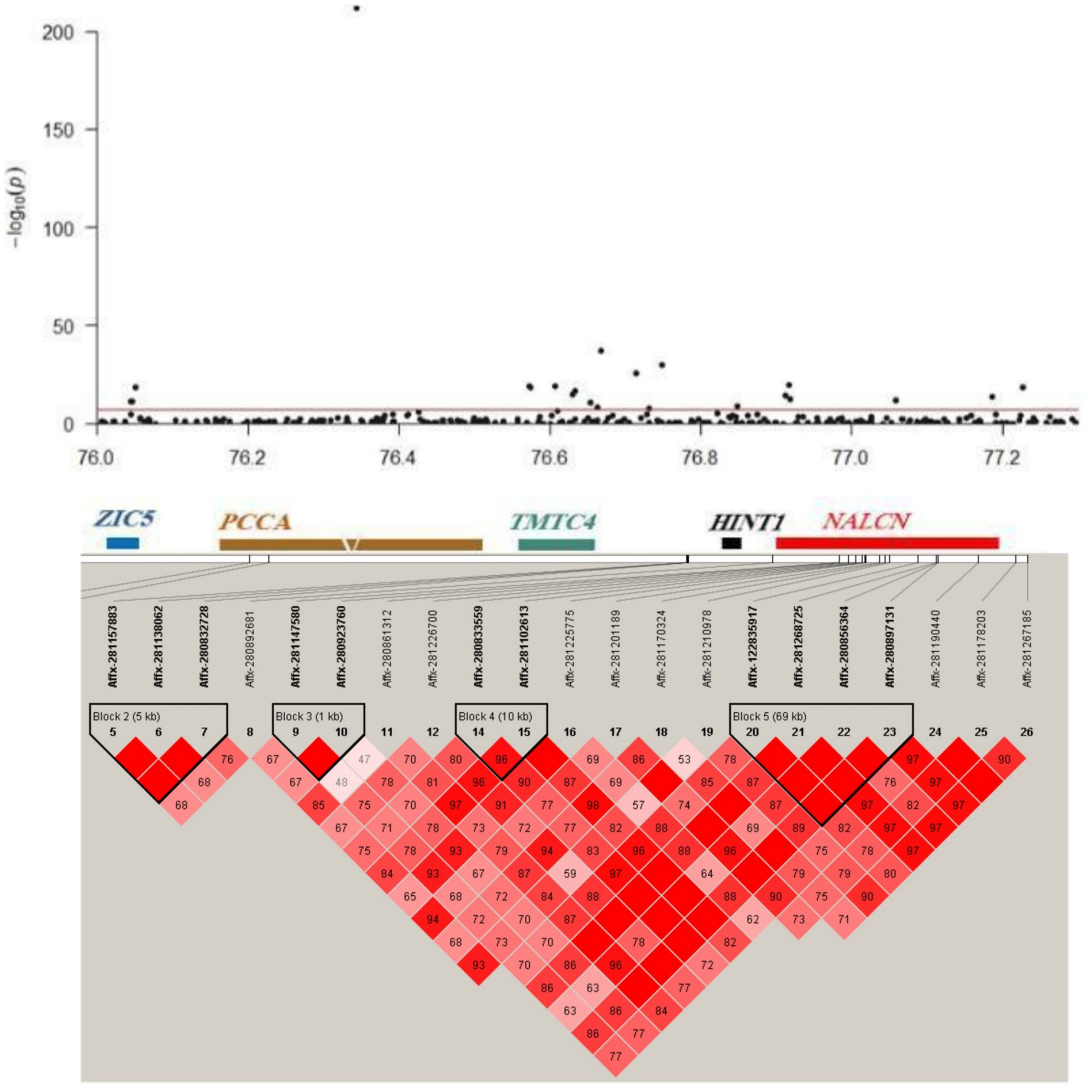
Linkage disequilibrium (LD) blocks of the novel loci detected using multi-trait GWAS. Manhattan plot (top) and LD plot (bottom) of the 76.04–77.23 Mb genomic region on chromosome 10.

**Figure 4:**
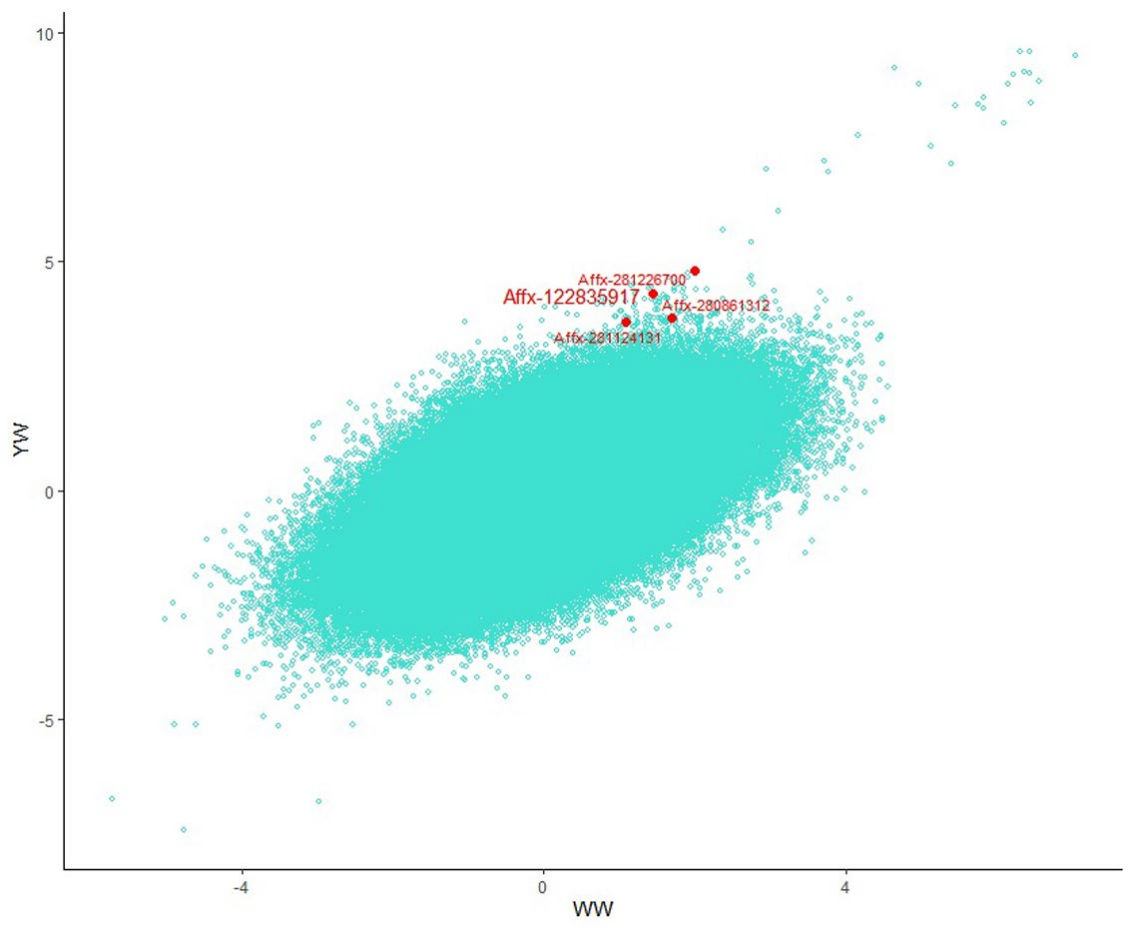
Scatter plot comparing all Beta/SE values for the two traits across the genome. Novel loci detected by muti-trait GWAS are marked at the edges of the plot.

Using multi-trait GWAS, we identified a new genomic region at 76.04–77.23 Mb on chromosome 10 containing 22 significant SNP loci (Figure 3). These loci were missed in the single-trait GWAS analysis. However, the observed odds ratios of the two traits showed sufficient deviations, and the statistical significance could only be identified after considering the joint statistics of the two phenotypes (Figure 4). These results showed that multi-trait GWAS can increase statistical power and complement the results of single-trait GWAS. Of those significant SNPs, Affx-280892681, located at 76.34 Mb on the *PCCA* (Propionyl-CoA carboxylase subunit alpha) gene, was the most significant loci (*p* = 1.43 × 10^−212^). But all near loci with it is not significant, we speculate that it is a false positive loci related to body weight. In this region, there are 12 significant SNP loci at 76.40–76.90 Mb were strongly linkage disequilibrium (r^2^ = 0.89). The Affx-122835917 SNP, located near the *HINT1* gene, was associated with BW (*p* = 9.22 × 10^−10^).

### 3.4. Expression profiles of candidate genes across multiple tissues

To validate biological function of these candidate genes in this study, we explored RNA-Seq data of multi-tissues of juvenile and adult sheep. We found there were 13 and 6 genes expressed in all 17 tissues of juvenile and adult stages, respectively. Of these genes, *ARID1B* (AT-Rich Interaction Domain 1B), *DNM1L* (Dynamin 1 Like), *FANCM* (Fanconi anemia complementation group M), *HINT1* (Histidine Triad Nucleotide Binding Protein 1), and *ZCCHC17* (Zinc Finger CCHC-Type Containing 17) were expressed in all development stages, and the expression of *HINT1* gene was highest. According STRING Interaction Network database, the fatty acid-binding protein family gene, including *FABP3*, *FABP5*, *FABP7* proteins, were interacted with *HINT1*. So we deem the *HINT1* gene might play important role involving in body weight.

The expression patterns each gene varied in different tissues of different development stages. We calculated index of tissue specificity each genes. The results shown *ASB11* (Ankyrin Repeat And SOCS Box Containing 11), *KIAA1549L* (KIAA1549 Like), *GPR143*, *UNC79* (Unc-79 Homolog, NALCN Channel Complex Subunit) were specifically expressed in muscle biceps, brain, hypothalamus, and pituitary tissues of juvenile and adult stages, respectively.

By difference expression analysis, we found *ASB11* gene was significantly higher expressed in muscle biceps tissue of juvenile sheep (*p* = 6.69 × 10^−4^), and *GPR143* gene was significantly higher expressed in hypothalamus tissue of adult sheep (*p* = 1.64 × 10^−4^).

## 4. Discussion

Multi-trait GWAS is usually used to detect QTLs associated with multiple traits, when there is a covariance between traits. The higher the genetic and phenotypic correlation between traits, the higher is the statistical power of multi-trait GWAS. In this study, our results showed that the genetic correlation between weaning weight and yearling weight was 0.73 and Singh et al. (29) found the genetic correlation between the two traits in marwari sheep was 0.56. These findings indicate that there is a positive genetic correlation between weaning weight and yearling weight in sheep. To improve the power of GWAS results, we conducted bi-trait GWAS for two correlated traits. In comparison single trait GWAS, we yielded 93 novel SNPs related to these traits. Using the same strategy, Zhou et al. (30) conducted multi-trait GWASs for chest, abdominal, and waist circumferences in Duroc Pig populations and detected four additional SNPs. Yan et al. (31) identified 16 novel loci associated with hematological traits in the White Duroc × Erhualian F2 resource population; Bolormaa et al. (32) discovered that multi-trait analysis improves the detection of polymorphic QTLs for 32 traits in beef cattle. Together with our findings, these results show that multi-trait GWAS can complement single-trait GWAS results and thus increase the statistical power of GWAS, when there is genetic correlation between different traits.

Very few studies have examined SNPs or QTLs related to weaning weight and yearling weight in sheep. According to the SheepQTLdb database (as of April 25, 2023), there are 11 QTLs and 4 QTLs related to weaning weight and yearling weight in sheep, respectively, based on QTL mapping or GWAS. These QTLs are distributed on OAR2–OAR4, OAR7, OAR9, OAR15, OAR19, and OAR24 (1, 9, 10, 33). Our study expanded this list considerably, identifying 148 SNPs that are significantly correlated with weaning weight and yearling weight through a combination of single- and multi-trait GWAS. However, we found that the candidate genetic markers of body weight identified in this study were less consistent than those reported from previous GWAS. This difference may be due to differences in the genetic background and breeds of sheep or their size and population structure. Differences in the detection platforms or algorithms used for analysis and random or technical errors in some analyses may have also contributed to these differences. Nevertheless, this suggests that many important genetic markers and candidate genes of weight traits in the sheep genome remain to be discovered.

In this study, we performed a genome-wide association study of body weights of 218 ewes. This is a small study. Although, many researchers insist on a large sample, and “the larger the sample, the more reliable is the result” is their dictum. Multiple problems have been cited with the studies on a small sample (34, 35). Whether based on a small sample or a large sample, no single study is considered conclusive. A large number of small studies can be done easily in different condition. Anderson and Vingrys (36) argued that small samples may be enough to show the presence of an effect but not for estimating the effect size. If most of small studies point toward the same direction, a possibly robust conclusion can be drawn through a meta-analysis. Animal experiments can be done in highly controlled conditions to nearly eliminate all the confounders, thus it may be used small sample to establish the cause-effect relationship (37). When more confounders were under control, sufficient power is achieved with a smaller sample. This study was only few confounders, such as birth year of ewes. So far, there are many examples exist of useful studies on small samples. For example, significant associations with body weight, growth-related and body conformation traits were identified by GWAS in 96 Baluchi sheep (38), 69 Egyptian Barki sheep (39), 150 Dazu Black goats (40), respectively. Furthermore, we also deem that estimated effects, confidence intervals and exact *p* values should be considered when interpreting a study’s results, but only sample size (41), and some exact methods of statistical analysis may help in reaching more valid conclusions for small sample size.

In contrast to long-held notions whereby single genes were believed to encode single functions, most genes are now recognized to have multiple qualitatively distinct functions. This phenomenon is termed pleiotropy (42). Pleiotropy is defined as a condition in which a single locus affects two or more distinct phenotypic traits (43, 44). It is very common phenomenon in nature for pleiotropism. The present study also found an interesting phenomenon where both the *GPR143* and *SHROOM2* genes were significantly associated with weaning weight and yearling weight. Hence, these two genes appear to be pleiotropic. Lu et al. (1) also revealed that *GPR143* and *SHROOM2* are associated with birth weight, weaning weight, yearling weight, and adult weight in sheep, which is consistent with the results of the present study. Zhang et al. and Jahejo et al. also found *SHROOM2* gene is closely associated with tibial cartilage dysplasia (45, 46). It is of great significance to make a profound study of the pleiotropy so that it can reveal common genetic mechanisms between closely related phenotypes, as well as the molecular functions of genes. So, further functional data are required for the validation of these findings.

Due to high conservation across species, the identified genes related to body weight traits in humans and other animals may also be important for sheep growth and development. In this study, we found that some of these genes, including *ARID1B, ASB11, DNM1L, HNF4A* (Hepatocyte Nuclear Factor 4 Alpha), *MKX* (Mohawk Homeobox)*, PKIB* (CAMP-Dependent Protein Kinase Inhibitor Beta)*, TBL1X* and *TMTC4* (Transmembrane O-Mannosyltransferase Targeting Cadherins 4) may be related to sheep body weight traits (Table 4). Liu et al. (47) found that *ARID1B* mutations are strongly associated with growth and weight traits in humans. *ASB11* is a major regulator of human embryonic and adult regenerative myogenesis (48). Increased expression levels of the *DNM1L* proteins may correlate with the degree of weight gain, and is closely related to the development of obesity (49–52). *HNF4A* mutations are associated with a considerable increase in birth weight and macrosomia, and the gene acts in the intestine and kidney to promote white adipose tissue energy storage (53–56). *MKX* is a potential regulator of brown adipose tissue development associated with obesity-related metabolic dysfunction in children (57). *PKIB* plays a central role in human obesity and metabolism (58, 59). *TBL1X* mainly plays an important role in maintaining precursor adipocytes in an undifferentiated state by inhibiting adipogenesis (60). Ma et al. (61) showed that *TMTC4* is significantly related to the formation of human skeletal muscle. From the above elementary description of the candidate genes, we find some of them are more or less associated with muscle development and body weight in different species, which allows us to predict the genes might take part in similar processes in sheep genome. Subsequent studies, such as functional verification, will be done in the candidate genes, which could ultimately reveal the causal mutations underlying body weigh traits in sheep.

**Table 4:** Basic functions of the identified genes.

Gene ID	Position(kb)	Full name	Function
*ARID1B*	OAR8:80106814–80481520	AT-rich interaction domain 1B	Linked to human growth disorders (47)
*ASB11*	OARX:12753078–12782477	Ankyrin Repeat And SOCS Box Containing 11	A major regulator of human embryonic and adult regenerative myogenesis (48)
*DNM1L*	OAR3:181841759–181886894	Dynamin 1 Like	Related to the development of obesity (49–52)
*HNF4A*	OAR13:72383636–72412129	Hepatocyte Nuclear Factor 4 Alpha	Associated with a considerable increase in birth weight and macrosomia (53–56)
*MKX*	OAR13:35933444–35998635	Mohawk Homeobox	A potential regulator of brown adipose tissue (57)
*PKIB*	OAR8:15590257–15710072	CAMP-Dependent Protein Kinase Inhibitor Beta	Related to obesity and metabolism in humans (58, 59)
*TBL1X*	OARX:6723480–6835914	Transducin Beta Like 1 X-Linked	Inhibiting adipogenesis (60)
*TMTC4*	OAR10:76587978–76636800	Transmembrane O-mannosyltransferase targeting cadherins 4	Skeletal muscle formation (61)

## 5. Conclusion

In this study, we identified 148 significant SNPs related to weaning weight or yearling weight based on single-trait and multi-trait GWAS. Two important chromosomal regions were discovered, including the 6.74–7.04 Mb interval on chromosome X and the 76.04–77.23 Mb interval on OAR10. Our results suggest that multi-trait GWAS is a powerful statistical tool for identifying novel loci missed by conventional single-trait GWAS. Incorporated transcript expression data of candidate genes, *HINT1*, *ASB11* and *GPR143* genes may involve in sheep body weight. This study show multi-omic anlaysis is a valuable strategy identifying candidate genes. Moreover, they provide key insights into the genetic determinants of weight traits in sheep.

## Data availability statement

The datasets presented in this study can be found in online repositories. The names of the repository/repositories and accession number(s) can be found at: https://ngdc.cncb.ac.cn/; PRJCA002639, GVM000068.

## Ethics statement

The animal study was reviewed and approved by the Experimental Animal Care and Use Committee of the Xinjiang Academy of Agricultural and Reclamation Sciences (Shihezi, China, approval number: XJNKKXY-AEP-039, January 22, 2012). The Northeast Agricultural University (Harbin, China) Animal Care and Treatment Committee (IACUCNEAU20150616). Written informed consent was obtained from the owners for the participation of their animals in this study.

## Author contributions

HY, ZW, PZ, and YL conceived the study. YY and QY were involved in the acquisition of data. YL and JG performed all data analysis. ZW, YL, PZ, and HY drafted the manuscript. YG and CZ contributed to the writing and editing. All authors contributed to the article and approved the submitted version.

## Funding

This work was supported by Young and middle-aged scientific and technological innovation leading talent plan of the Xinjiang Production and Construction Corps (2019CB019), the Open Project of State Key Laboratory of Sheep Genetic Improvement and Healthy Production (MYSKLKF202001), the National Key R&D Program of China (No. 2021YFD1300903-2), the State Key Laboratory of Sheep Genetic Improvement and Healthy Production (No. MYSKLKF201902), the National Natural Science Foundation of China (Nos. 32070571 and 32160770), Agricultural Science and Technology Innovation Project of the Xinjiang Production and Construction Corps (No. NCG202211) and Major Scientific and Technological Project of the Xinjiang Production and Construction Corps (No. 2017AA006-02).

## Conflict of interest

The authors declare that the research was conducted in the absence of any commercial or financial relationships that could be construed as a potential conflict of interest.

## Publisher’s note

All claims expressed in this article are solely those of the authors and do not necessarily represent those of their affiliated organizations, or those of the publisher, the editors and the reviewers. Any product that may be evaluated in this article, or claim that may be made by its manufacturer, is not guaranteed or endorsed by the publisher.

## Footnotes

^1^https://www.ensembl.org/Ovis_aries/Info/Index

^2^http://www.animalgenome.org/cgi-bin/QTLdb/OA/browse

^3^http://www.bioinformatics.babraham.ac.uk/projects/fastqc/
